# An fMRI Study of the Effects of Vibroacoustic Stimulation on Functional Connectivity in Patients with Insomnia

**DOI:** 10.1155/2020/7846914

**Published:** 2020-02-04

**Authors:** George Zabrecky, Shiva Shahrampour, Cutler Whitely, Mahdi Alizadeh, Chris Conklin, Nancy Wintering, Karl Doghramji, Tingting Zhan, Feroze Mohamed, Andrew Newberg, Daniel Monti

**Affiliations:** ^1^Department of Integrative Medicine and Nutritional Sciences, Thomas Jefferson University, Philadelphia, PA 19107, USA; ^2^Department of Radiology, Thomas Jefferson University, Philadelphia, PA 19107, USA; ^3^Sleep Disorders Center, Department of Psychiatry and Human Behavior, Thomas Jefferson University, Philadelphia, PA 19107, USA; ^4^Department of Biostatistics, Thomas Jefferson University, Philadelphia, PA 19107, USA

## Abstract

**Background:**

It is well known that vibratory and auditory stimuli from vehicles such as cars and trains can help induce sleep. More recent literature suggests that specific types of vibratory and acoustic stimulation might help promote sleep, but this has not been tested with neuroimaging. Thus, the purpose of this study was to observe the effects of vibroacoustic stimulation (providing both vibratory and auditory stimuli) on functional connectivity changes in the brain using resting state functional magnetic resonance imaging (rs-fMRI), and compare these changes to improvements in sleep in patients with insomnia.

**Methods:**

For this study, 30 patients with insomnia were randomly assigned to receive one month of a vibroacoustic stimulation or be placed in a waitlist control. Patients were evaluated pre- and postprogram with qualitative sleep questionnaires and measurement of sleep duration with an actigraphy watch. In addition, patients underwent rs-fMRI to assess functional connectivity.

**Results:**

The results demonstrated that those patients receiving the vibroacoustic stimulation had significant improvements in measured sleep minutes as well as in scores on the Insomnia Severity Index questionnaire. In addition, significant changes were noted in functional connectivity in association with the vermis, cerebellar hemispheres, thalamus, sensorimotor area, nucleus accumbens, and prefrontal cortex.

**Conclusions:**

The results of this study show that vibroacoustic stimulation alters the brain's functional connectivity as well as improves sleep in patients with insomnia.

## 1. Introduction

Insomnia is a major, chronic problem affecting up to 30% of all people and causing significant loss of function and productivity [1, 2]. There are many causes of insomnia, but essentially all of them are associated with altered neuronal activity, particularly in structures such as the thalamus, prefrontal cortex, parietal lobe, brain stem, cerebellum, and caudate nucleus. Studies suggest that altered neuronal activity, particularly a persistent hyperarousal state, causes insomnia [3] Thus, primary insomnia may be a final common pathway that develops from the interplay between an inborn vulnerability for an imbalance between arousing and sleep-inducing brain activity combined with various external and internal stressors that perpetuates a mechanism of hyperarousal in the brain [3]. Prolonged insomnia may also result in altered neurophysiological changes that are associated with subsequent cognitive or emotional problems [4, 5]. Impaired sleep quality or quantity, insomnia, may be caused by neurophysiological changes such as alterations in neuronal communication between structures such as the thalamus and various cortical regions [6], and that may be reflected in functional neuroimaging, though this is an underinvestigated area. Several authors have suggested that therapies that target the prevalent hyperarousal state might be useful in the management of insomnia [3, 7]. Thus, the current study focuses on the potential causal hyperarousal mechanism of insomnia by attempting to modify this pathophysiological process through external auditory and vibratory stimuli designed to entrain the brain waves and improve sleep (see Section 2.3). In addition, the goal of this study is to observe changes in functional connectivity that might help demonstrate the specific brain structures that are ultimately affected by these external stimuli.

Many patients with insomnia are placed on medications to promote sleep, but these can result in a distorted sleep architecture and are associated with the potential for side effects. Certain sleep-promoting medications, such as the benzodiazepine hypnotics, can diminish slow-wave sleep proportions, thus altering sleep architecture [8]. Nonpharmacological approaches might be advantageous, particularly if they are able to improve brain functional connectivity without the adverse effects of these drugs.

One approach for improving sleep and restoring natural brain sleep architecture has been through the use of vibratory and auditory stimulation with the goal of reducing the hyperarousal mechanisms leading to insomnia [9]. It has long been noted that many people fall asleep in cars and trains that typically produce a vibration along with a sound stimulus [10]. Although the vibrations of running cars or trains typically include more than 10 mm amplitude in waveform, it is almost impossible to sleep under an artificially produced continuous large amplitude stimulus in large part due to motion sickness. In addition, a vibration with a frequency of 2.0 Hz or more may not contribute to induce sleep [11]. Thus, for each direction, the maximum amplitude and frequency are defined as 10 mm and 2.0 Hz, respectively. A related study exploring the use of a vibration-producing bed for sleep found that improved sleep resulted from low-amplitude vibration in both the vertical and horizontal directions. The average sleep latencies were comparably improved for both vertical and horizontal excitation at amplitudes of 2.4 mm to 7.5 mm [12].

Various forms of vibratory and acoustic stimuli to the body have been observed to subjectively improve how one feels [13, 14]. Auditory stimulation appears to alter brain wave patterns and can enhance delta patterns (an important component of the sleep state) on electroencephalography (EEG) [15]. Another study showed that auditory stimulation enhances sleep spindles, thus enhancing sleep [16]. In addition, several small pilot studies utilized an audiovisual stimulation in patients with chronic insomnia and found significant improvements in insomnia symptoms and sleep quality [17, 18]. Another approach utilized music developed from EEG patterns and similarly found substantial improvements in sleep patterns in patients with insomnia [19]. Individual studies as well as systematic reviews and meta-analyses have shown that listening to music has also been shown to be helpful in promoting sleep [20–23].

For the present study, we decided to utilize a program that includes both auditory and vibratory stimulation (i.e., vibroacoustic stimulation) to try to promote sleep using a mechanism similar to those described above primarily targeting increased delta waves. We hypothesized that if such an approach improves sleep, this will be reflected by alterations in functional connectivity among structures that are involved in the regulation of sleep. Several scholars have recommended the use of neuroimaging techniques to better evaluate therapeutic approaches in sleep disorders [24]. Thus, the purpose of this project is to evaluate whether vibroacoustic stimulation has a physiological effect on functional connectivity in the brain and to correlate such changes with changes in insomnia levels.

Functional MRI studies have revealed changes in several brain regions in patients with insomnia. One study of 21 older adults with primary insomnia showed that during cognitive tasks, patients had hypoactivation in the left prefrontal cortex and left inferior frontal gyrus, in comparison to good sleepers [25]. After six weeks of multimodal nonpharmacological therapy, activation was partially restored in the medial prefrontal cortex during the category fluency task, and in the inferior frontal gyrus during the letter fluency task. Other fMRI studies comparing functional connectivity in insomnia patients to that in controls revealed an association with the insular cortex, middle frontal gyrus, prefrontal cortex, parietal lobe (particularly the precuneus), and head of the caudate nucleus [26, 27]. Additionally, functional connectivity between the cerebellum and various cortical structures is altered in patients with insomnia [28].

Assessing function using fluorodeoxyglucose positron emission tomography (PET), Nofzinger et al. showed that primary insomnia patients had lower waking metabolism, compared to healthy controls, in cortical (bilateral frontal, left superior temporal, and parietal cortices) and subcortical regions (thalamus and brainstem reticular formation) [29]. In addition, these research studies found evidence for prefrontal deactivation in patients with insomnia. While the relationship between functional connectivity and cerebral metabolism remains unclear in insomnia patients, the findings of both types of studies demonstrate that there are substantial neurophysiological effects of insomnia. Based on the abovementioned imaging studies, we hypothesized that a vibratory and auditory stimulation program might directly affect areas of the brain that receive such stimuli (including the cerebellum, sensorimotor areas, and auditory cortex) and indirectly affect areas of the brain that support cognitive or emotional function (such as the prefrontal cortex, parietal lobe, amygdala, and nucleus accumbens).

Given the above-described findings, the purpose of this pilot study was to evaluate if a vibroacoustic stimulation program alters brain physiology in insomnia patients as measured by functional connectivity using resting state fMRI (rs-fMRI) and whether such changes are associated with improvements in sleep. Thus, the current study used resting Blood Oxygen Level Dependent (BOLD) functional connectivity analysis in patients initially and then after completing participation in the program of vibroacoustic stimulation. We also observed changes in minutes slept and perceived sleep quality after completing the vibroacoustic stimulation program.

## 2. Materials and Methods

### 2.1. Overview

All patients had the study explained in detail, were allowed to ask any questions, and then signed the informed consent form that was approved by the Thomas Jefferson University Institutional Review Board. This study did not meet the criteria for listing on clinicaltrials.gov since it did not involve testing an experimental medication or device. We recruited 36 patients who met the inclusion criteria for the study. Patients had to have a history of insomnia disorder for the past 3 months, as defined by the Diagnostic and Statistical Manual-5 criteria (patients were allowed to have initial or middle insomnia since the therapeutic intervention is intended to be used at the onset of sleep and during midsleep awakenings); be aged 18-80 years old; have no other preexisting and active significant medical, neurological, or psychological disorders; have no previous brain surgery or intracranial abnormalities that may complicate interpretation of the brain scans; could not be pregnant or lactating; have nothing to inhibit or hinder lying still in the scanner (i.e., claustrophobia or weight > 350 pounds); and have no metal in their body or other reason that they could not undergo magnetic resonance imaging. Patients were allowed to have minor, stable health problems that should have no substantial effect on cerebral blood flow (i.e., controlled hypertension, controlled thyroid condition, or controlled diabetes). Furthermore, patients were allowed to be taking medications or supplements at the initial intake, but must have been on a stable dose regimen for at least 1 month and remain on that regimen throughout their participation in the study unless a change was required for medical reasons. In the overall cohort, 3 patients were taking thyroid medication, 2 patients were taking cholesterol medication, 2 patients were taking antihypertensive medications, 1 patient was taking bupropion, and 1 patient was taking amphetamine/dextroamphetamine for many years.

### 2.2. Subjects

Subjects who met the inclusion criteria underwent an initial sleep evaluation (see below) along with resting state BOLD fMRI. Subjects were then randomized in a 2 : 1 manner into an active group or a waitlist control group. Of the 39 patients recruited, 30 patients completed the study (2 patients could not tolerate the scanning, 4 withdrew due to scheduling conflicts, and 3 dropped out for unspecified reasons). Demographic information and the pre- and postsleep data on the subjects who completed the study in both groups are provided in Table 1.

The vibroacoustic stimulation group received auditory and vibratory stimulation for one month and then underwent the same imaging and sleep evaluation as performed during the initial evaluation. The waitlist control group was evaluated initially and then had the same imaging and sleep evaluation one month later. The waitlist group was then offered the opportunity to receive the auditory and vibratory stimulation program for one month at no charge, and without additional scanning.

### 2.3. Auditory and Vibratory Stimulation

For the vibroacoustic stimulation, we utilized the Theracoustic VibrAcoustic Wellness System™ 3.0 (see http://www.theracoustic.com/). The overall goal as reported by the manufacturer is to help entrain the brain's electrical wave frequencies to those that are associated with sleep. Several studies have shown that weak sine-wave electric fields help to entrain slow oscillation in vitro [30], and this type of slow oscillation closely resembles the activity pattern during slow-wave sleep [31, 32]. These researchers concluded that weak, constant, sine-wave fields enhance and entrain the slow oscillation. In a similar manner, the program used in the present study system delivers auditory and vibratory stimulation using primarily a sine wave pattern. The specific program used included two components.

The first component involved vibroacoustic stimuli in which patients came to the Marcus Institute of Integrative Health to receive a 24-minute vibroacoustic program targeted to deliver sine wave-based auditory and vibratory stimuli at the frequency of theta waves associated with a state of relaxation (8-10 Hz) with amplitudes between 0 and 5 mm. This was performed using a combination multichannel harmonic and vibroacoustic digital audio system incorporated into a comfortable lounge chair. The second, audio component, is a 60-minute audio program designed to help subjects entrain their brain in the delta wave range of frequencies. The audio program was delivered through a high-fidelity digital audio player with noise-suppressing stereo headphones or earbuds used by the subjects. These sessions are self-administered while the subject is prone in bed. The audio session moves initially from the 12 Hz range to a theta state range (between 4 and 10 Hz), and then to the delta state range (1-4 Hz) which is sustained for the rest of the hour. The self-administered at-home audio sessions were 60-minute duration programs and targeted at the delta brainwave state of 1-4 Hz.

During the study, subjects were instructed to utilize the “in-house” vibroacoustic program two times per week for one month and to use the “at-home” auditory program each night for that same month as they went to bed. The basis for the 30-day duration for the vibroacoustic intervention was to allow for sufficient time to observe an effect, and also because significant clinical effects using this same system were observed over a similar time period [33]. Subjects were also provided diaries to record their nightly compliance with the program. Subjects were instructed to continue using the programs until their second, postintervention, fMRI scan.

### 2.4. Sleep Measures

Sleep evaluations occurred prior to the vibroacoustic stimulation program and then again after 4 weeks of receiving the program. To evaluate the sleep status, subjects wore an actigraphy monitor (Philips) for 5 consecutive nights. This was performed during the week prior to using the vibroacoustic program and then again during the week following the use of the program. During the recording time, we focused primarily on minutes slept per night. In addition to the actigraphy measurement, all patients completed the self-report-based Insomnia Severity Index (ISI) [34] at both the pre- and postmeasurement times.

### 2.5. MRI Measures and Analysis

The MR imaging was performed in a Siemens mMR 3 T PET-MRI scanner using a standard 12-channel head coil. On the initial (pre-) and follow-up (post-) scans, the following MRI sequences were used to acquire brain images of various contrasts. After a localizer scan, a T1 Magnetization-Prepared Rapid Gradient Echo (MPRAGE) sequence was used to collect high-resolution structural images of the brain. The following imaging parameters were used: Field of View (FOV) = 25.2 cm; voxel size = 0.5 × 0.5 × 1.0 mm^3^; TR = 1600 ms; TE = 2.46 ms; slice thickness = 1 mm; number of slices = 176; flip angle = 9; and acquisition time = 446 s. Next, a resting state BOLD scan was collected using an Echo Planar Imaging (EPI) sequence to examine intrinsic functional connectivity of the brain regions. The following imaging parameters were used: FOV = 23.6 cm; voxel size = 3 × 3 × 4 mm^3^; TR = 2.0 s; TE = 30 ms; slice thickness = 4 mm; number of slices = 34; number of volumes = 180; and acquisition time = 366 s. During rs-fMRI, the subjects were instructed to close their eyes, keep their heads still, and rest quietly without thinking about anything in particular for 5 minutes. Total scan time was approximately 40 minutes.

In an effort to uniquely describe the communication between resting state networks without the influence of noise contaminants, a specialized analysis pipeline is required. This process starts with spatial preprocessing using Statistical Parametric Mapping (SPM) 12 (Wellcome Trust Centre for Neuroimaging at UCL) along with the CONN toolbox which is an open source Matlab- (Mathworks, Inc.: web.conn-toolbox.org) based cross-platform imaging software for the computation, display, and analysis of functional connectivity data [35]. The resting state connectivity analysis pipeline includes preprocessing of fMRI data using the CONN component-based noise correction CompCor strategy (the CompCor method takes into account the influence of a voxel-specific combination of various estimated noise sources such as cardiac and respiratory effects to eliminate artefacts in the estimated connectivity measure), ROI (region of interest) mask creation, time series extraction of ROIs, computing connectivity matrices, and first- and second-level analyses. After the experiment information is set up in the CONN toolbox, the preprocessing is initiated to remove possible confounds in the BOLD signal. The general preprocessing pipeline used in CONN is as follows: (a) realignment—fMRI data are realigned with the primary aim of removing motion artefacts in the fMRI time series (in this step, the first image is selected as reference and the subsequent images are realigned to the first one using a series of rigid body spatial transformation); (b) slice-timing correction—corrects the differences in image acquisition time between slices; (c) outlier detection; (d) coregistration—puts functional data and the structural data in the same space; (e) segmentation of structural MRI in grey matter, white matter, and CSF—applies a tissue probability map to put the structural MRI into a standard template space; (f) normalization—applies forward deformations from the segmentation step to put the functional data into a standard space; and (g) smoothing—in this step, the signal is averaged to reduce the noise, which helps to increase the signal-to-noise ratio (SNR). After the preprocessing is complete, a seed-based functional connectivity group analysis is performed. This method assists in finding within-group differences through selected ROIs. Regions of interests (ROIs) are defined and extracted using combined FSL Harvard-Oxford and AAL atlases. Resting state functional connectivity was calculated using two-sided bivariate correlations. Next, significant rest-state functional connectivity differences among subjects were evaluated before and after vibroacoustic or control conditions using ROI-to-ROI analysis with initial threshold connections of *P*-uncorrected (*ρ* < 0.05). This connection is defined as the bivariate correlation coefficients between two ROIs and BOLD time series. The time series are calculated by averaging the voxel time series across all voxels within each ROI. We then used a post hoc correction for multiple comparison using the false discovery rate based on the specific regions we targeted in the analysis with a threshold set at *p* < 0.05.

The target ROIs were selected by the brain areas which were hypothesized to be involved with insomnia and also vibroacoustic stimulation. Specifically, we evaluated the thalamus, prefrontal cortex, parietal lobe, brain stem, vermis and cerebellar hemispheres, sensorimotor region, auditory cortex, amygdala, nucleus accumbens, and caudate nucleus. All the ROI regions are in the CONN toolbox and are separated as left and right as specified in the Results (Table 2) in which we have identified which laterality has had an effect. In general, the toolbox provides a series of default and predefined regions of interest (ROI) that were loaded automatically for brain parcellation for cortical, subcortical, and cerebellar areas from the FSL Harvard-Oxford Atlas. In this default atlas, the vermis and cerebellar hemispheres are defined separately. From the available regions, we focused only on the regions specified above as these were targeted based on our initial hypothesis of areas that were likely involved.

### 2.6. Additional Statistical Measures

#### 2.6.1. Randomization

Randomization occurred via a 2 : 1 ratio using the method of random permuted blocks with random block sizes without stratification. Subjects were randomized into the vibroacoustic stimulation program or the waitlist control group.

Statistical analysis of the Insomnia Severity Index and minutes slept were performed using a linear mixed-effects model. Variable selection based on second-order Akaike information criterion were performed using the R package MuMIn [36, 37]. To clarify, the resting state analysis and effect of clinical changes are performed using two separate statistical test and models—a *t*-test and a linear mixed-effects model, respectively.

## 3. Results

### 3.1. Clinical Changes

Thirty patients completed the study (see Table 1 for patient group information) who had been randomly assigned to either the vibroacoustic group (*N* = 19) or the waitlist control group (*N* = 11). There were no significant differences in the two patient groups based on Fisher's exact test or *t*-test, where appropriate; at baseline with regard variables such as age, gender, and duration of sleep problems, there were no significant differences as well (*p* > 0.05). There was no significant difference between ISI scores of the two groups at baseline (*p* = 0.62); however, there was a significant difference in minutes slept (*p* = 0.02). There were significant improvements in the ISI score in those subjects undergoing the vibroacoustic stimulation compared to the controls (see Table 1). Specifically, the active group had a reduction of -3.1 in their ISI score (*p* < 0.001 compared to previbroacoustic stimulation). Furthermore, at the conclusion of the active group, 9 out of 19 patients had an ISI less than 7, whereas 3 out of 11 patients in the control group had an ISI less than 7.

In addition, the active group had a mean increase of 30.6 minutes per night compared to previbroacoustic stimulation (*p* = 0.001) as well as when compared to the control group response (*p* < 0.001). This was not accounted for by an increased time in bed which was not significantly different between the two groups both in the pre- and postevaluation state. Furthermore, there was a mean increase of 14 minutes in bed for the control group and 6 minutes in bed for the vibroacoustic group (*p* = 0.20).

### 3.2. rs-fMRI Results

When comparing the group that underwent vibroacoustic stimulation to the control group, there were several significant differences in functional connectivity as shown in Table 2. These reveal significant changes between the vermis and both the sensorimotor and auditory cortex, the right thalamus and right caudate, the cerebellar hemispheres and the sensorimotor cortex, and the right prefrontal cortex and the right nucleus accumbens. It should be noted that when comparing the vibroacoustic stimulation group to the control group at baseline, there were no significant differences in functional connectivity in the regions we targeted in our analysis. None of the other regions of interest revealed statistically significant changes in functional connectivity between the two groups.

## 4. Discussion

In the present study, the use of both auditory and vibratory stimulation resulted in improved sleep measures and altered functional connectivity in brain structures previously described as involved with insomnia and sleep regulation. Clinically, there was a statistically significant improvement in both the minutes slept, using an actigraphy monitor, and in the Insomnia Severity Index scores, in the group receiving the vibroacoustic stimulation program compared to waitlist controls. Additionally, although not part of the formal data collection, patients in the initial waitlist group, who then used the vibroacoustic stimulation program, generally reported positive responses in terms of improvement in sleep amount and quality. These findings suggest that vibroacoustic stimulation may improve actual sleep measures as well as the subjective measure of sleep quality reported by patients. Given the results from this initial proof-of-concept study, we plan to perform studies of longer duration and also assess the long-term impact of vibroacoustic stimulation on sleep measures beyond a one-month period.

This study is consistent with several other studies performed with both the current technology as well as a related vibration-producing bed. For example, the results from these studies showed benefits for improving sleep for a maximum amplitude and frequency of 10 mm and 2.0 Hz, respectively, in both the vertical and horizontal directions [11, 12]. Earlier studies, using the same system as in this study, in 76 patients with addiction disorders over a 30-day intervention period, reported improvements in sleep measures, as well as stress levels and cravings, anxiety, fear, and anger [33]. Thus, the current study corroborated these earlier reports showing that the vibroacoustic stimulation program improves sleep in patients with insomnia.

The present study also helps elucidate the mechanism by which a program of auditory and vibratory stimulation might improve sleep. The functional connectivity analysis shows several significant differences between the active vibroacoustic stimulation group and the control group. The right thalamus and right caudate have increased functional connectivity. The thalamus and caudate appear to be involved in both sleep and wakefulness, since the caudate is involved with arousal mechanisms in conjunction with the prefrontal cortex [38] and the thalamus is a central relay for many cortical-cortical networks and cortical-subcortical networks and might contribute to a persistent hyperarousal state hypothesized to result in insomnia [39]. It is interesting that the right hemisphere has been more implicated as abnormal in patients with insomnia, although the exact reason behind this asymmetry is unclear. Several studies have shown a right predominant loss of white matter integrity in patients with insomnia [40]. Studies in healthy volunteers have reported left-hemisphere predominance during wakefulness and right-hemisphere predominance during sleep [41]. Additionally, it has been hypothesized that the right hemisphere might have higher vulnerability to insomnia-related disruption [42], but future research will be required to better elucidate such a mechanism.

One study showed the thalamus to be affected in response to sleep-related sounds after cognitive-behavioral therapy in patients with psychophysiological insomnia [43]. Another study showed that effective cognitive-behavioral therapy for insomnia resulted in decreased functional connectivity in the thalamus and parietal cortex, putamen and motor cortices, and the amygdala and lingual gyrus, but increased functional connectivity between the caudate and supramarginal gyrus, the pallidum and orbitofrontal cortex, and the hippocampus and frontal/parietal gyri [44]. While our study did not observe the exact results as in those studies mentioned above, there were functional connectivity changes in some of the same brain structures such as the thalamus and caudate, and in some different structures such as the prefrontal cortex and nucleus accumbens. Some of the differences between our results and the other studies were in part due to focusing on different structures, since we only targeted the thalamus, prefrontal cortex, parietal lobe, brain stem, vermis and cerebellar hemispheres, sensorimotor region, auditory cortex, amygdala, and caudate nucleus. These regions have been observed to be affected in patients with insomnia, in addition to several other structures such as the insula or middle frontal gyrus [26, 28, 45].

An important question is how brain changes associated with improved sleep can be differentiated from the direct effects of vibroacoustic therapy. We hypothesized that the auditory and vibratory stimulation program would result in different types of central nervous system changes. In fact, two areas we were particularly interested in regarding the effect of vibratory stimulation would be the sensorimotor area and the cerebellum which would most likely be affected such stimuli. The results supported this hypothesis since both areas demonstrated significant changes in functional connectivity. On the other hand, several studies have already implicated the sensorimotor areas [45] and cerebellum more directly with insomnia itself [46, 47], including the vermis [48]. Furthermore, the spinocerebellum, comprised of the vermis and also parts of the cerebellar hemispheres, receives proprioceptive input from the dorsal columns of the spinal cord, as well as from the auditory and visual systems. Thus, we expected the observed changes in the cerebellar hemispheres and vermis to be associated with a program that is based upon vibratory and auditory stimulation.

We also did not observe changes in several structures that previously have been implicated in insomnia. Specifically, there were no significant changes in the parietal lobe or the limbic structures such as the amygdala that had been reported in prior studies [25, 29]. It is possible that these areas are associated with cognitive or affective aspects of sleep loss such as poorer cognition or increased anxiety and depression. Since the patients involved in our study did not report significant baseline problems with anxiety and depression symptoms as determined by their initial observations reported by the patients during screening and also as assessed with standard measures (i.e., Spielberger Anxiety Scale and Beck Depression Index), this may explain why we did not observe changes in these clinical measures or potential associated brain regions. Future studies can try to better delineate changes that are more specific to the vibratory and auditory stimulation program compared to changes that are more specific to improvements in sleep itself (e.g., improved cognition or emotional status).

Limitations of the present study include a small sample size. Although randomized, the vibroacoustic group did have a significantly lower mean value for minutes slept at baseline even though the mean ISI scores were not different. This baseline reduction could account for a greater upward response to the vibroacoustic intervention even though there was no significant change in the control group. Future studies will need to enroll a larger number of subjects to determine if such a program would be effective in a wide variety of patient populations suffering from impaired sleep. Specifically, it would be important to evaluate whether such a system would be useful in patients with sleep problems associated with psychiatric conditions including anxiety or depression as well as medical conditions such as cancer or heart disease. In addition, a larger sample would allow for a better determination of how variables such as age, gender, and duration of insomnia problem factor into the analysis model. Regarding the imaging data, we focused our analysis on specific structures involved with both insomnia as well as auditory and vibratory stimulation, but future studies might explore other brain regions, although the results from such an analysis might be limited by multiple comparisons. We utilized approved equipment for monitoring sleep but more formal sleep studies, including those that measure EEG changes, might be useful to better measure the effects of auditory and vibratory stimulation on sleep patterns. Additionally, we compared vibroacoustic stimulation to a waitlist control, but a more active control group might provide a clearer determination of the effectiveness of this program. Furthermore, the waitlist group could have been treated and then tested later on and used to replicate the results of the vibroacoustic group in a separate sample, or the vibroacoustic group could have been tested again at a follow-up one month later to see whether changes in their functional connectivity and improvement in insomnia were stable or not. This would have added to the significance of the current results, but limitations in funding allowed only analysis of the initial two time points. Finally, it will be important for future studies to compare such a program to other approaches that might help improve sleep including nonpharmacological methods such as meditation-based programs, as well as pharmacological approaches using either approved sleep medications or natural supplements.

## 5. Conclusion

This preliminary neuroimaging study suggests that future studies are warranted to better explore whether a program of vibroacoustic stimulation is effective in patients with insomnia as well as in those patients with impaired sleep associated with other health problems.

## Figures and Tables

**Table 1:** Demographic information and sleep data pre- and postvibroacoustic stimulation or waitlist period.

	Vibroacoustic group	Control group
Gender (male/female)	9/10	7/4
Age (mean ± SD)	43.3 ± 19.6	40.8 ± 13.6
Age range	(27 to 75 years)	(21 to 83 years)
ISI measure pre (mean ± SD)	13.1 ± 5.7	12.7 ± 4.8
ISI measure post (mean ± SD)	8.6 ± 4.7^∗^	11.7 ± 5.5
Minutes slept pre (mean ± SD)	431 ± 46	467 ± 29
Minutes slept post (mean ± SD)	479 ± 62^∗^	470 ± 27

^∗^ *p* value < 0.001 when compared between pre- and postvalues for the vibroacoustic stimulation group compared to controls.

**Table 2:** Results show functional connectivity differences between the two groups for the regions that survived post hoc correction for multiple comparisons using the false discovery rate method (FDR-corrected *p* values provided). *x*, *y*, and *z* coordinates of each target ROI are also shown, representing the centroid of the ROI. A two-sample *t*-test is calculated for resting state functional connectivity between the groups for the following ROIs.

Insomnia group (vibroacoustic stimulation vs. control group/pre vs. post)			
Brain structures	FDR-corrected *p*	*t*-test	*x*, *y*, *z* coordinates
Vermis-sensorimotor	0.009	-4.16	Sensorimotor superior network: (-0.073, -30.535, and 67.405)
Vermis-R auditory cortex	0.0435	-3.30	Auditory cortex (R): (46.110, -17.401, and 6.961)
R thalamus-R caudate	0.032	+3.26	R caudate: (13.301, 10.010, and 10.49)
R cerebellar hemisphere-R sensorimotor	0.0375	-2.97	Sensorimotor (R) network: (56.386, -9.868, and 28.818)
R nucleus accumbens-R PFC	0.0402	-2.96	Frontoparietal PFC (R) network: (-43.116, 33.186, and 28.244)
L cerebellar hemisphere-L sensorimotor	0.0355	-2.93	Sensorimotor (L) network: (-55.467, -12.364, and 29.489)

## Data Availability

The data used to support the findings of this study are available from the corresponding author upon request.

## Abbreviations

BOLD: Blood oxygen level dependent
cm: Centimeter
EEG: Electroencephalography
EPI: Echo planar imaging
FOV: Field of view
FWHM: Full width half maximum
ISI: Insomnia Severity Index
L: Left
mm: Millimeter
MPRAGE: Magnetization-prepared rapid gradient Echo
MRI: Magnetic resonance imaging
ms: Milliseconds
PET: Positron emission tomography
PFC: Prefrontal cortex
R: Right
rs-fMRI: Resting state functional magnetic resonance imaging
s: Seconds
SD: Standard deviation
SPM: Statistical parametric mapping
ROI: Region of interest
TE: Echo time
TR: Repetition time.
